# Circular Skin Lesions Mimicking Blunt Trauma: A Forensic Case of Cupping Therapy

**DOI:** 10.7759/cureus.87240

**Published:** 2025-07-03

**Authors:** Ikuto Takeuchi, Motoo Yoshimiya, Atsushi Ueda, Yu Kakimoto

**Affiliations:** 1 Department of Forensic Medicine, Tokai University School of Medicine, Isehara, JPN

**Keywords:** bruise mimics, cupping therapy, differential diagnosis of abuse, forensic pathology, skin lesions

## Abstract

Skin findings provide important diagnostic clues in both clinical and forensic practice, often serving as an initial trigger for evaluating the possibility of physical assault or child abuse. However, not all discolorations that resemble bruises result from blunt trauma. Cupping therapy, a traditional complementary treatment widely practiced in East Asia, can produce purplish skin discolorations caused by superficial capillary rupture from localized negative pressure. We present a forensic autopsy case of a man who died following a fall from height. During postmortem examination, multiple circular purplish discolorations were observed on his upper back, initially raising suspicion of blunt trauma or assault. However, postmortem dissection revealed no hemorrhage in the subcutaneous fat or muscle tissue beneath the lesions, and further investigation identified a history of recent cupping therapy. This case highlights the importance of recognizing cultural practices such as cupping therapy that may mimic traumatic injuries and underscores the need for careful differentiation in both forensic and clinical evaluations to avoid diagnostic errors.

## Introduction

Observation of skin findings provides valuable diagnostic information in both clinical and forensic practice. External examination can offer crucial clues when evaluating injuries. It often serves as the first indication to consider possibilities such as physical assault or child abuse. However, not all skin discolorations that look like bruises are caused by blunt trauma. Various mechanisms can produce similar appearances, sometimes leading to diagnostic confusion [1-3].

Cupping therapy is a form of traditional complementary medicine that remains widely practiced, particularly in East Asia. Cupping therapy is a traditional practice with historical roots in many cultures, including ancient Egypt, China, and the Middle East. It involves the application of cups to create negative pressure, which is believed to stimulate circulation and promote healing. Numerous clinical reports have documented its use for musculoskeletal pain relief and other indications [4-6]. The procedure involves applying localized negative pressure to the skin using heated cups or mechanical suction devices. This creates characteristic purplish discolorations caused by superficial capillary rupture (small broken blood vessels under the skin). These marks may closely resemble bruises caused by blunt trauma [1,3,7].

In this report, we present a forensic autopsy case in which circular discolorations on the back, initially suggestive of contusions, were ultimately determined to result from cupping therapy. This case highlights the importance of recognizing alternative etiologies for skin findings that mimic traumatic injuries. This report aims to highlight the importance of recognizing culturally related skin findings that can be misinterpreted as traumatic injuries during forensic examinations.

## Case presentation

A man in his 30s was found collapsed in a public park near a 15-meter-high bridge by a groundskeeper. Although there were no eyewitnesses, the location and circumstances suggested a fall from height. Upon arrival at the emergency department, he presented with hypotension and impaired consciousness. Computed tomography revealed a pelvic fracture and intra-abdominal bleeding. Despite intensive resuscitative efforts, he died shortly after arrival due to hemorrhagic shock.

**Figure 1:**
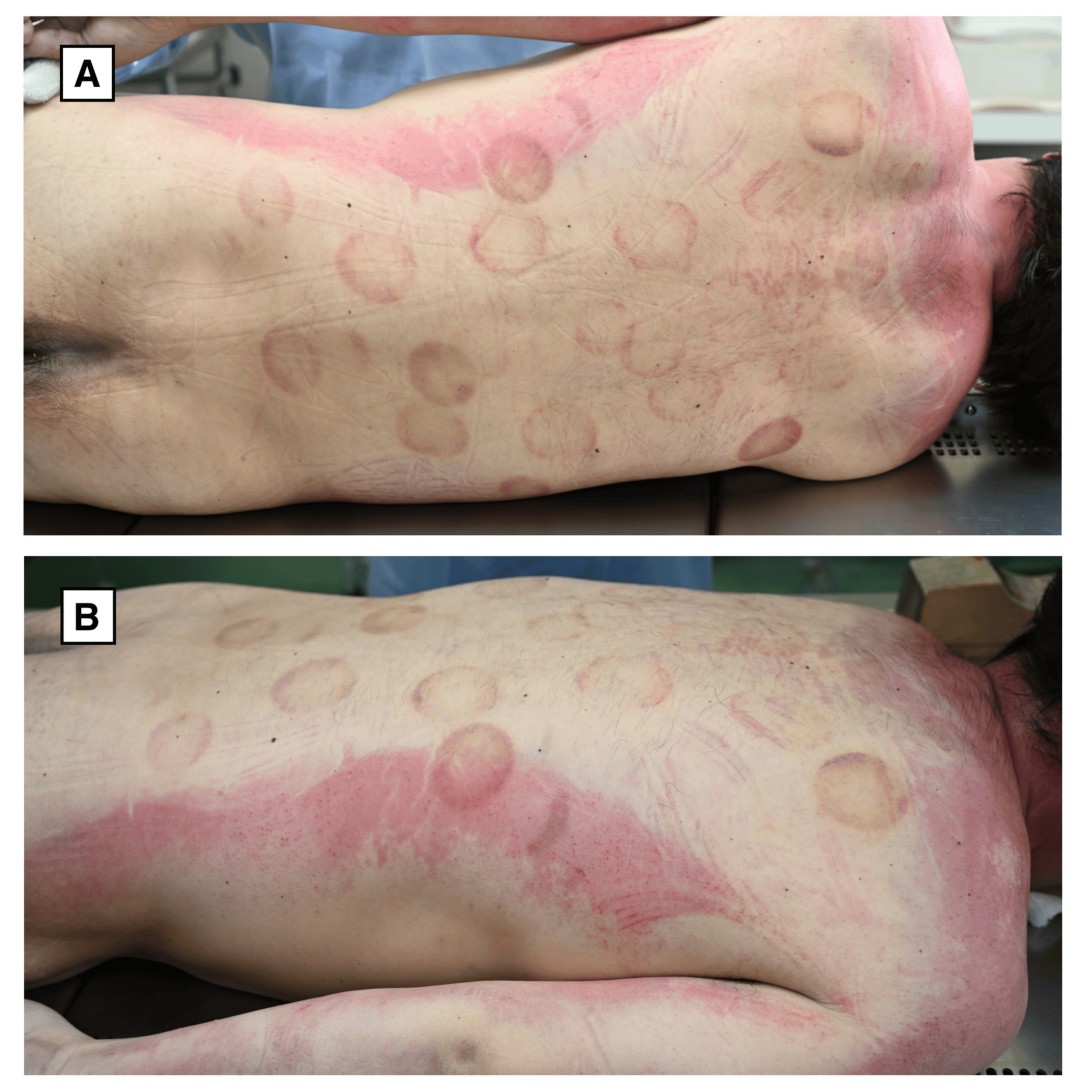
Circular skin discolorations on the back A) Posterior view showing multiple, well-demarcated circular purplish discolorations distributed across the back from the shoulders to the lower spine. B) Lateral view highlighting the same lesions from a side angle, confirming their bilateral symmetry and superficial appearance.

Postmortem examination confirmed fatal pelvic trauma consistent with a high-impact fall. In addition to these injuries, approximately 25 well-demarcated circular purplish discolorations, each measuring approximately 3-5 cm in diameter, were observed across the back, extending from the shoulders to the lower lumbar region (Figure 1A and Figure 1B). 

Apart from these circular discolorations, no other remarkable external injuries were noted on the skin surface. Initially, these lesions raised concern for blunt force injuries caused by physical abuse or assault.

According to information obtained during the police investigation, the deceased had been regularly undergoing cupping therapy at a local clinic prior to the incident.

## Discussion

**Figure 2:**
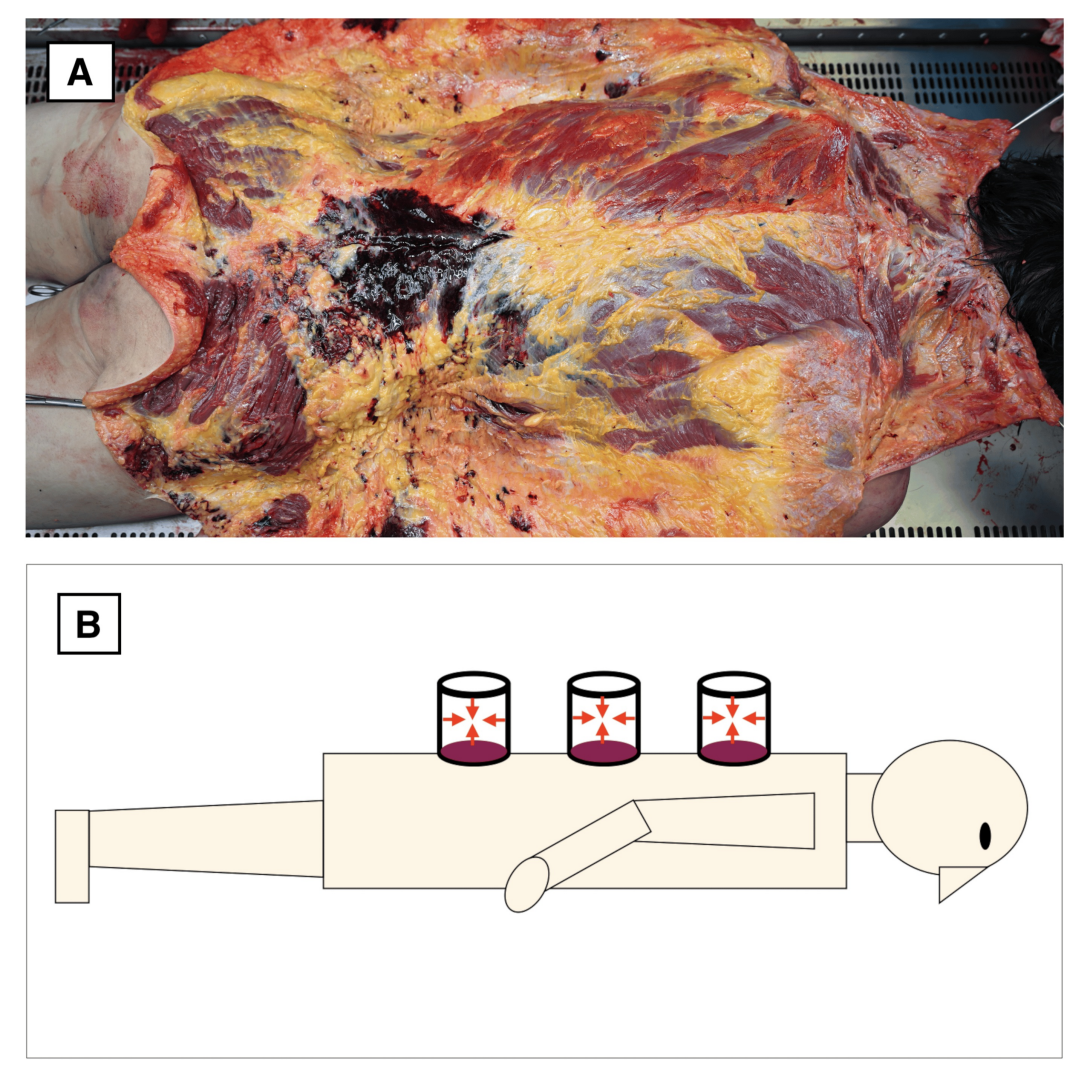
Comparison between postmortem findings and cupping therapy mechanism A) Subcutaneous tissue dissection revealing no evidence of hemorrhage or deep tissue damage beneath the circular skin lesions. B) Illustration of the cupping therapy mechanism: A cup is applied to the skin to create negative pressure, resulting in characteristic circular marks caused by superficial blood pooling without underlying trauma. Image Credit: Authors

In this case, postmortem dissection revealed no associated hemorrhage in the subcutaneous fat or muscle tissue beneath the lesions (Figure 2A), effectively ruling out deep tissue trauma.

The regular, circular shape of the lesions and the confirmed history of cupping therapy further supported their non-traumatic etiology.

Cupping therapy, also known as fire cupping or vacuum cupping, is a traditional healing practice widely used for musculoskeletal pain relief. It involves creating localized negative pressure on the skin using heated glass or plastic cups, or mechanical suction devices (Figure 2B) [6]. This negative pressure draws blood into the superficial dermal layers, resulting in round purplish skin discolorations that may resemble bruises.

Unlike traumatic contusions (bruises caused by impact), which typically have irregular shapes and deeper soft tissue injury, cupping-induced lesions are well-demarcated, uniform in size, superficial, and often symmetrically distributed [8].

Differentiating between physical maltreatment and cupping practices is critical, as shown in pediatric forensic cases [7].

Adverse effects of dry cupping commonly include localized skin redness and purpura at the sites of application. In addition, when fire suction techniques are used to create negative pressure, there is a potential risk of thermal injury to the skin. Actual cases of burn injuries following cupping procedures have been reported, including pediatric cases misinterpreted as possible abuse [1,9]. Previous reports have described cases of cupping-related skin discolorations misinterpreted as signs of abuse, particularly in pediatric populations [5,7]. Although our case involved an adult, it demonstrates that similar diagnostic challenges can arise when patients are deceased or have impaired consciousness and are unable to provide information about recent cultural or therapeutic practices. In previously published cases with detailed descriptions of skin findings, the circular purplish discolorations measured approximately 3 cm in diameter, which is similar to the lesions observed in our case [5,9].

In addition to cupping, skin injuries resulting from other negative pressure devices, such as suction pumps, have been reported to mimic intentional injuries and raise concerns of possible abuse [2].

Importantly, skin findings from cultural healing practices, including cupping, skin scraping (Gua Sha), and moxibustion, can look like signs of physical abuse. Moxibustion is a traditional therapy that involves burning dried herbal material (moxa) near the skin surface to apply heat for therapeutic purposes. This resemblance can lead to diagnostic confusion [3]. In both forensic and clinical settings, such misinterpretations can result in inappropriate legal action or unnecessary interventions. Other medical devices, such as ECG electrode suction pads, can also produce circular marks that closely resemble bruises. Therefore, careful assessment of treatment history and any recent medical procedures is essential to avoid misinterpretation of skin findings, especially when the patient cannot provide information. Therefore, clinicians and forensic professionals must recognize these traditional practices and integrate cultural background, lesion morphology, and autopsy findings to achieve accurate interpretations.

## Conclusions

Cupping therapy may produce circular purplish skin discolorations that closely resemble bruises caused by blunt trauma, potentially leading to misinterpretation as signs of physical abuse or assault. Thorough forensic examination, including dissection when necessary, is essential to differentiate these superficial suction-induced lesions from true traumatic injuries. Clinicians and forensic experts must remain aware of cultural healing practices to avoid diagnostic errors and inappropriate legal or medical actions.

## References

[1] Focardi M, Gori V, Romanelli M (2024). "Mimics" of injuries from child abuse: case series and review of the literature. Children (Basel).

[2] Heath KJ, Byard RW (2015). Suction pump injuries mimicking child abuse. Forensic Sci Med Pathol.

[3] Look K, Look R (1997). Skin scraping, cupping, and moxibustion that may mimic physical abuse. J Forensic Sci.

[4] Cao H, Han M, Li X (2010). Clinical research evidence of cupping therapy in China: a systematic literature review. BMC Complement Altern Med.

[5] Cao H, Li X, Liu J (2012). An updated review of the efficacy of cupping therapy. PLoS One.

[6] Mehta P, Dhapte V (2015). Cupping therapy: a prudent remedy for a plethora of medical ailments. J Tradit Complement Med.

[7] Lupariello F, Coppo E, Cavecchia I, Bosco C, Bonaccurso L, Urbino A, Di Vella G (2020). Differential diagnosis between physical maltreatment and cupping practices in a suspected child abuse case. Forensic Sci Med Pathol.

[8] Lowe DT (2017). Cupping therapy: an analysis of the effects of suction on skin and the possible influence on human health. Complement Ther Clin Pract.

[9] Moura CC, Chaves ÉCL, Cardoso AC, Nogueira DA, Corrêa HP, Chianca TC (2018). Cupping therapy and chronic back pain: systematic review and meta-analysis. Rev Lat Am Enfermagem.

